# The relationship between systemic disorders and anatomical outcomes after Descemet membrane endothelial keratoplasty

**DOI:** 10.5935/0004-2749.20220087

**Published:** 2025-02-11

**Authors:** Husna Topcu, Burcin Kepez Yildiz, Yusuf Yildirim, Alper Agca

**Affiliations:** 1 Department of Ophthalmology, Inebolu State Hospital, Kastamonu, Turkey; 2 University of Health Sciences, Beyoglu Eye Training and Research Hospital, Istanbul, Turkey; 3 Department of Ophthalmology, Dunya Goz Hospital, Istanbul, Turkey

**Keywords:** Descemet membrane, Endothelial keratoplasty, Diabetes mellitus, Endothelial cell count, Hypertension, Membrana de Descemet, Ceratoplastia endotelial, Diabetes mellitus, Contagem de células endoteliais, Hipertensão

## Abstract

**Purpose:**

The aim of this study was to investigate the association of anatomical outcomes and medications of patients with systemic diseases who underwent Descemet membrane endothelial keratoplasty with donor factors.

**Methods:**

Sixty nondiabetic donors of endothelial grafts and 60 patients who underwent operation by a single surgeon were included in this retrospective study. The patients’ data, including the presence of diabetes mellitus and hypertension, antidiabetic-antihypertensive medications, and intracameral tamponades and anatomical outcomes, were recorded. The donor data were obtained from eye bank records.

**Results:**

Eighteen patients had type 2 diabetes mellitus (30%) and 34 had hypertension (56.6%). Among the patients with diabetes mellitus, 13 were receiving a single-agent antidiabetic drug, 4 were receiving dual oral antidiabetic therapy, and 1 was receiving insulin therapy. Among the hypertensive patients, 11 had monotherapy and 23 had dual antihypertensive therapy. Postoperatively, 35 patients (58.3%) had an endothelial attachment, 8 (13.3%) received reinjection, 7 (11.7%) required re-Descemet membrane endothelial keratoplasty, and 10 (16.7%) underwent penetrating keratoplasty. The mean donor age was 51.2 ± 14.1 years. The most common cause of donor death was cardiopulmonary arrest (36/60 cases; 60.0%). Regression analysis revealed that the presence of diabetes mellitus significantly disrupted graft attachment (p=0.034), while the presence of hypertension, antidiabetic and antihypertensive medication use, and the type of tamponade used in the patients, and the age, sex, cause of death, and specular endothelial cell count of donors were not statistically significantly associated with graft attachment (p>0.05).

**Conclusion:**

In this study, the anatomical outcomes of Descemet membrane endothelial keratoplasty surgery were affected by recipient and donor factors. The presence of diabetes mellitus in the recipient significantly negatively affected graft attachment.

## Abstract (pt)

**Objetivo:**

Investigar a associação de desfechos ana tômicos com doenças sistêmicas e medicamentos em casos submetidos à ceratoplastia endotelial da membrana de Descemet e fatores relativos aos doadores.

**Métodos:**

Foram incluídos neste estudo retrospectivo enxertos obtidos de doadores não diabéticos e 60 casos operados por um único cirurgião. Foram registrados os dados dos casos, incluindo a presença de diabetes mellitus e hipertensão, medicamentos antidiabéticos e anti-hipertensivos, tamponamentos intracamerais e desfechos anatômicos. Os dados dos doadores foram obtidos dos prontuários do banco de olhos.

**Resultados:**

Dezoito casos tinham diabetes mellitus tipo 2 (30%) e 34 tinham hipertensão (56,6%). Entre os casos de diabetes mellitus, 13 estavam em uso de uma medicação antidiabética de agente único, 4 estavam em terapia antidiabética oral dupla e 1 estava em insulinoterapia. Entre os hipertensos, 11 estavam em monoterapia e 23 em terapia anti-hipertensiva dupla. No pós-operatório, 35 pacientes (58,3%) submeteram-se a uma fixação endotelial, enquanto 8 casos (13,3%) receberam reinjeção, 7 casos (11,7%) necessitaram de ceratoplastia endotelial da membrana de Descemet e 10 casos (16,7%) foram submetidos a uma ceratoplastia penetrante. A média de idade dos doadores foi de 51,2 ± 14,1 anos. A causa mais comum de morte do doador foi parada cardiorrespiratória (36/60 casos; 60,0%). A análise de regressão revelou que a presença de diabetes mellitus causa distúrbios significativos na fixação do enxerto (p=0,034), enquanto a presença de hipertensão, o uso de medicamentos antidiabéticos e anti-hipertensivos, o tipo de tamponamento usado, a idade, o sexo, a causa da morte e a contagem de células endoteliais especulares dos doadores não demonstraram associações estatisticamente significativas com a fixação do enxerto (p>0,05).

**Conclusão:**

Os resultados anatômicos da cirurgia de ceratoplastia endotelial da membrana de Descemet são afetados por fatores do receptor e do doador. A presença de diabetes mellitus no receptor teve um significativo impacto negativo na fixação do enxerto.

## INTRODUCTION

Descemet membrane endothelial keratoplasty (DMEK) has been successfully used in cases of corneal dystrophy and pseudophakic bullous keratopathy as a transplantation technique with an extremely low risk of immunological graft rejection, rapid healing, and satisfactory visual results^(1-4)^. In 2006, this technique was first described by Gerrit Melles as the separation of healthy donor endothelial-Descemet’s membrane complex from the corneal stroma and transplantation of the complex to the recipient^(5,6)^.

In addition to its various well-known ocular manifestations, diabetes mellitus (DM) also has effects such as delayed wound healing in the cornea and reduced corneal sensitivity. The latest studies further clarified how DM affects DMEK surgery from both the recipients’ and donors’ perspectives. The presence of DM in the donor was demonstrated to increase the risk of failure in endothelial graft preparation and lead to a prolonged graft preparation time^(7)^. On the other hand, the presence of DM in the recipient was reported to be associated with an increased rate of reinjection of tamponade in the anterior chamber (re-bubble rate)^(8)^. Vianna et al. identified other systemic diseases associated with tissue preparation failure, including hypertension (HT), hypercholesterolemia, obesity, and DM disease duration, in the list of factors for consideration^(9)^. In the present study, we aimed to investigate the association of the anatomical outcomes of DMEK surgery using endothelial grafts obtained from the corneas of nondiabetic donors with the systemic diseases and medical treatments of recipients.

## METHODS

After a retrospective search in the medical records of patients who underwent DMEK surgery in Beyoglu Eye Training and Research Hospital between 2017 and 2018, 60 patients who underwent operation by a single surgeon were included in the study. The study protocol was approved by the Clinical Studies Ethics Committee of UHS Istanbul Taksim Training and Research Hospital (approval No. 138), and the study was conducted in accordance with the ethical principles state in the Declaration of Helsinki.

Chronic diseases such as DM and HT, and the antidiabetic and antihypertensive treatment protocols of the patients were retrieved from the medical database records. The ophthalmologic histories and examination findings of the patients were recorded. Their data on perioperative endothelial graft preparation, type of intracameral tamponade used, and intraoperative complications were retrieved from their surgical records. Surgical success based on the anatomical attachment status of the endothelium and the procedures performed in the patients who required interventions were recorded during the postoperative follow-ups. Patients with a history of previous trabeculectomy, drainage implant, or vitrectomy and any ocular trauma were excluded from the study.

The donor data were obtained from the eye bank of the Beyoglu Eye Training and Research Hospital. The corneas of the donors with a diagnosis of DM were excluded from the study. The age, sex, cause of death, and specular endothelial cell count of the donors were recorded.

### Surgical technique

All the patients underwent operation by the same experienced surgeon (A.A.). The endothelial-Descemet’s membrane complex was perioperatively stripped from the donor cornea and transplanted by the surgeon. All the surgeries were performed with the same surgical technique for DMEK as previously described by Kruse et al^(10)^. To minimize the risk of pupillary block, intraoperative iridectomy was performed. The surgeon used 100% air or 20% sulfur hexafluoride (SF6) gas in accordance with his prediction of the patient’s compliance for maintaining a supine position. If the patient was uncompliant, the surgeon preferred air because of the shorter resorption period and vice versa for SF6. All the patients were instructed to maintain a supine position during the early postoperative period when air/gas bubble was still present in the anterior chamber. All the patients remained in the hospital for 7 days postoperatively.

Achievement of endothelial attachment in a single session was considered an anatomical success criterion, whereas cases with readministration of tamponade (reinjection), re-DMEK, or penetrating keratoplasty (PKP) were considered failure.

### Statistical analyses

Data were analyzed using the IBM SPSS Statistics 18 software (SPSS Inc. 1989, 2010). The conformity of the continuous variables to a normal distribution was analyzed using the Kolmogorov-Smirnov test, and the continuous variables showed a normal distribution. The categorical variables used in the study are presented as frequency and percentage, whereas the continuous variables are presented as mean and standard deviation because they showed a normal distribution. The categorical variables were analyzed using the chi-square significance test and Fisher exact test, and a *t* test was used to compare the means of the continuous variables. Moreover, the potential factors identified in the univariate analyses were subsequently analyzed using a multiple logistic regression model. A p value of <0.05 was considered statistically significant.

## RESULTS

Among the included patients, 41 were female and 19 were male. The mean age of the patients was 68.3 ± 10.6 years (range, 43-89 years). Forty-six patients (76.7%) had pseudophakic bullous keratopathy, of whom 3 had an anterior chamber intraocular lens. Fuchs endothelial dystrophy was present in 14 patients (23.3%). Seven patients (50%) with Fuchs endothelial dystrophy underwent combined cataract surgery. Eighteen patients (30%) had DM, 34 (56.7%) had HT, and 14 (23.3%) had both DM and HT. Eight patients (13.3%) had no systemic disease or history of chronic medication use. Among the diabetic patients, 13 (72.2%) were receiving a single-agent antidiabetic therapy, 4 (22.2%) were receiving dual oral antidiabetic therapy, and one (5.6%) was receiving insulin therapy. Among the oral antidiabetics, metformin was the most commonly used agent in both the monotherapy and dual therapy groups. Ten patients (55.6%) used metformin as monotherapy, and 4 (22.4%) used it in a dual therapy. Only one patient was receiving an insulin glargine and glulisine regimen. Among the hypertensive recipients, 11 (32.4%) and 23 (67.6%) were receiving monotherapy and dual therapy, respectively. The angiotensin-converting enzyme inhibitors (16 patients, 47.0%) and thiazide diuretics (19 patients, 55.8%) were the most commonly used antihypertensives as monotherapy or dual therapy.

The tamponade agent administered into the anterior chamber during surgery was air in 17 patients (28.3%) and 20% SF6 gas in 43 patients (71.7%). None of the patients developed complications during the surgery. After operation, 35 patients (58.3%) had an endothelial attachment, 8 (13.3%) required reinjection, 7 (11.7%) required re-DMEK, and 10 (16.7%) required PKP. Eleven of the 18 patients (61.1%) with a diagnosis of DM did not achieve anatomical success, and this was statistically significant (p=0.046). Of the patients, 5 received reinjection, 2 underwent re-DMEK, and 4 received PKP. Sixteen (47.0%) of the 34 patients with HT did not have endothelial attachment. Of the patients with failure, 5 received reinjection, 5 underwent re-DMEK, and 6 received PKP. While anatomical success was achieved in 8 of the patients with both DM and HT diagnosis, 6 patients had anatomical failure, but this was not statistically significant (p=0.223). Of the patients with failure with both DM and HT, 3 received reinjection, 2 underwent re-DMEK, and 3 received PKP.

Donor data obtained from the eye bank showed a mean donor age of 51.2 ± 14.1 years (range, 39-74 years). Twenty-two donors were female, and 38 were male. The most common cause of death among the donors was cardiopulmonary arrest (60.0%). The mean specular endothelial cell count of the donors was 2916.45 ± 364.83 cells/mm^2^ (range, 1989.00-3774.00 cells/mm^2^). No significant association was observed between anatomical success and the donor factors. Data on various factors related to anatomical success are presented in table 1. No significant association was found between anatomical success and antidiabetic and antihypertensive treatment protocols (Table 2).

**Table 1:** Association of anatomical success with recipient and donor characteristics

	Success (n=35)	Failure (n=25)	Total (n = 60)	p value
Sex, η (%) Female Male	26 (74.3) 9 (25.7)	15 (60.0) 10(40.0)	41 (68.3) 19(31.7)	0.241
DM, n (%) No Yes	28 (80.0) 7 (20.0)	14(56.0) 11 (44.0)	42 (70.0) 18(30.0)	**0.046**
HT, n (%) No Yes	17(48.6) 18(51.4)	9 (36.0) 16 (64.0)	26 (43.3) 34 (56.7)	0.333
Tamponade, n (%) Air SF6	12(34.3) 23 (65.7)	5 (20.0) 20 (80.0)	17(28.3) 43 (71.7)	0.226
Donor sex, n (%) Female Male	14(40.0) 21 (60.0)	8 (32.0) 17 (68.0)	22 (36.7) 38 (63.3)	0.526
Donor cause of death, n (%) Cardiac arrest	22 (62.9)	14(56.0)	36 (60.0)	0.726
Other Donor age^*^ Donor specular endothelial cell count^*^	13(37.1) 51.3 ± 14.3 2968.88 ± 396.71	11 (44.0) 51.0 ± 14.2 2843.04 ± 307.62	24 (40.0) 51.2 ± 14.1 2916.45 ± 364.83	0.921 0.190

* Mean ± SD.

**Table 2:** Association of anatomical success with antidiabetic and antihypertensive agents

	Success n (%)^*^	Failure n (%)	Total n (%)	p value
**Antidiabetic treatment (n=18)**				
Monotherapy	5(71.4)	8 (72.7)	13 (72.2)	0.387
Dual therapy	1 (14.3)	3 (27.3)	4 (22.2)	
Insulin	1 (14.3)	0 (0.0)	1 (5.6)	
	**Antihypertensive treatment (n**		**= 34)**	
Monotherapy	7 (38.9)	4 (25.0)	11 (32.4)	0.388
Dual therapy	11 (61.1)	12 (75.0)	23 (67.6)	

* Column percentage.

Then, a logistic regression model was applied to the data, and the results are presented in tables 3 and 4. We found that the presence of DM in the recipients was the most important risk factor in the univariate and multivariate models. On the basis of our results, the diabetic patients had a 1.4-fold higher risk of failure than the nondiabetic patients. In this model, age, sex, presence of HT, and type of tamponade used were not associated with anatomical success. No statistically significant association was found between donor age and anatomical success in our study, and the expected trend is that the risk of failure decreases in older donors^(11,12)^.

**Table 3:** Univariate logistic regression model

Variable	OR	95% Cl	p value
DM	3.143	1.001-9.870	0.05

CI= Confidence interval; OR= Odds ratio.

**Table 4:** Logistic regression model

Variable	OR	95% CI	p value
Age	0.008	0.951-1.069	0.787
Sex	0.958	0.714-9.517	0.147
DM	**1.498**	1.119-17.881	**0.034**
HT	0.229	0.333-4.752	0.735
Tamponade	0.817	0.534-9.595	0.267
Donor age	-0.023	0.931-1.026	0.354
Donor sex	0.290	0.349-5.116	0.672
Donor specular endothelial cell count	-0.001	0.997-1.000	0.141

* Nagelkerke *R*^^2^^=0.234

CI= Confidence interval; OR= Odds ratio.

## DISCUSSION

In recent years, with the increasing popularity of DMEK surgery, many studies reported various factors that affect anatomical and functional success. However, most of these studies focused on donor factors and investigated the association between endothelial graft preparation and donor characteristics. The present study, on the other hand, investigated whether the presence of DM and/or HT in recipients influenced the anatomical success of DMEK surgery using grafts obtained from the corneas of nondiabetic donors.

One of the first studies to investigate the effect of the presence of DM in donors who underwent DMEK surgery was performed by Greiner et al. by reviewing diabetic and nondiabetic donor records from two large eye banks in the United States^(7)^. Eye bank technicians experienced in preparing endothelial grafts observed that diabetic corneas are associated with a more difficult graft preparation process and a higher tendency of tearing. A study based on this observation revealed an endothelial graft preparation failure rate of 15.3%, which is 9.2-fold higher than that in nondiabetic corneas, when endothelial grafts were harvested from diabetic donors. The authors emphasized that the chronic hyperglycemia-induced molecular alterations in the posterior stroma-Descemet’s membrane interface may play a role in this failure. By using immunohistochemical methods, Schwarz et al. showed that the glycoproteins formed by the non-enzymatic glycation of proteins in the presence of high glucose levels led to strong adhesion between the posterior stroma and Descemet’s membrane^(13)^. In our study, in all the cases, the endothelial grafts were stripped manually by the surgeon preoperatively, and no complications occurred in any of the cases during graft preparation.

In their study that investigated the effect of DM on DMEK surgery from both donor and recipient perspectives, Price et al. found that the presence of DM in the donor increased the graft preparation failure rate by five-fold^(8)^. The presence of DM in the donor was not found to be related to the re-bubble and graft survival rates. However, the re-bubble rate was 33% in diabetic recipients receiving insulin therapy, 19% in diabetics not using insulin, and 17% in nondiabetic recipients (p=0.0023). They reported that the number of endothelial cells lost was higher in diabetic recipients than in nondiabetics during the 4-year graft follow-up period (p=0.02). Similarly to our study, Janson et al. evaluated results of DMEK using grafts from nondiabetic donors in diabetic recipients^(14)^. The patients were divided into three groups as follows: nondiabetic recipients, diabetic recipients receiving insulin therapy, and diabetic recipients not receiving insulin therapy. The graft survival rate was similar between all the groups, but the re-bubble rate was higher in the diabetic cases, although the difference did not reach statistical significance (p=0.15 and p=0.08, respectively). In our study, the multiple logistic regression model revealed that the failure rate increased by 1.4-fold in the presence of DM in the recipients (p=0.034). Eleven of the 18 patients with DM did not achieve endothelial attachment in a single session (p=0.046). Alteration of the aqueous profile and disruption of the blood-aqueous barrier with an increase in the levels of the inflammatory proteins might have negatively influenced the function of endothelial cells and graft survival in diabetic cases^(15,16)^. This hypothesis is supported by the clinical data of our study and the studies by Price and Janson^(8,14)^.

DM treatment may be divided into two groups, an insulin-based treatment group and a non-insulin-based treatment group. The most popular insulin-sensitizing agent is metformin, which is used as the first-line treatment for type 2 DM. Metformin was demonstrated to inhibit the development of age-related comorbidities such as cardiovascular disease, dementia, depression, and cancer^(17)^. In another study, oral metformin 2 g/day for >2 years was shown to decrease the risk of open-angle glaucoma in diabetic cases ^(18)^. In their study, Chocron et al. investigated the effect of metformin on endothelial cell count and found that the presence of DM was significantly associated with low endothelial cell counts but found no significant difference in endothelial count between diabetic patients treated with and those not treated with metformin^(19)^. In our study, metformin was the most commonly used antidiabetic agent in both the monotherapy and dual therapy groups. No significant association was found between surgical success and receiving monotherapy or dual therapy.

In their study, Vianna et al. investigated the association of factors such as DM and HT, hyperlipidemia or obesity, and tobacco use in donors with the success of endothelial graft preparation and found that these factors were associated with high failure rates^(9)^. DM and hyperlipidemia or obesity were statistically significantly associated with graft preparation failure (p=0.000028 and p=0.004, respectively). The authors emphasized that these factors cause the formation of toxic products in the presence of oxidative stress, such as the advanced glycation end products formed in diabetes, and may lead to alterations in the morphology of tissues. DM and HT can occur in the same individual simultaneosly. Williams et al. developed a 5-point DM rating scale to assess the severity of DM in donors^(20)^. In accordance with this scale, 1 point was added to the score when HT was present in the donor. Therefore, the presence of HT can be considered a factor that increases the effect of DM on the donor tissue preparation failure rate. In our study, we did not apply such rating to the recipients. However, most of the patients with diagnosed DM already had HT. In these cases, this co-incidence showed no significant effect on anatomical success (p=0.223). A study that compares diabetesand hypertension-only recipients may reveal the effect of DM more apparently. In our study, we in vestigated how the presence of HT, one of the abovemen tioned factors, affects surgical success. Sixteen of the 34 patients with HT did not achieve endothelial attachment in a single session, but this did not reach statistical significance. Twenty-three of these patients were taking dual antihypertensive treatments for blood pressure control. However, no significant association was observed between the treatment protocols and anatomical success.

Studies have reported that air and SF6 gas are effective and safe tamponade agents in DMEK surgery^(21,22)^. A longer duration of SF6 in the anterior chamber was found to be associated with a lower re-bubble rate and less graft detachment^(23)^. Air tamponade has also been reported to be effective in a large patient series, with low complication rates^(24)^. As no standard approach has been established for choosing the appropriate tamponade for each patient, the choice of tamponade may vary depending on the surgeon’s decision and the patient’s compliance in maintaining a supine position. In our study, no significant difference was found between the type of tamponade and anatomical success (p=0.226).

Age is another commonly investigated donor factor. Studies have reported that the unfolding time of the graft in the anterior chamber is longer, stripping off the endothelial-Descemet’s membrane complex is more difficult, and the number of endothelial cells lost is higher when using the corneas of young donors^(8,11,12)^. In our study, the mean donor age was 51.21 ± 14.18 years, and the multiple logistic regression analysis revealed no significant association between anatomical failure and donor age (p=0.354).

Our study has some limitations owing to its retrospective nature, including the small number of cases, similar numbers of diabetic recipients receiving and those not receiving insulin therapy, and the inability to identify blood HbA1C levels. We could have evaluated the association between recipients at earlier and more advanced stages of diabetes and surgical success from a broader perspective if these data had been available.

We observed that the presence of DM was associated with surgical failure in this study, in which we evaluated the success of DMEK surgery on the basis of recipient factors. Longer-term clinical studies with larger numbers of cases are necessary to better understand the magnitude of this association.
